# CBP22, a Novel Bacteriocin Isolated from *Clostridium butyricum* ZJU-F1, Protects against LPS-Induced Intestinal Injury through Maintaining the Tight Junction Complex

**DOI:** 10.1155/2021/8032125

**Published:** 2021-06-01

**Authors:** Tenghao Wang, Jie Fu, Xiao Xiao, Zeqing Lu, Fengqin Wang, Mingliang Jin, Yizhen Wang, Xin Zong

**Affiliations:** ^1^Key Laboratory of Molecular Animal Nutrition, Ministry of Education, College of Animal Sciences, Zhejiang University, Hangzhou, China; ^2^Key Laboratory of Animal Nutrition and Feed Science in Eastern China, Ministry of Agriculture, Hangzhou, Zhejiang Province, China; ^3^Zhejiang Qinglian Food Co., Ltd., Jiaxing, China

## Abstract

A novel bacteriocin secreted by *Clostridium butyricum* ZJU-F1 was isolated using ammonium sulfate fractionation, cation exchange chromatography, affinity chromatography, and reverse-phase high-performance liquid chromatography (RP-HPLC). The bacteriocin, named CBP22, contained 22 amino acids with the sequence PSAWQITKCAGSIAWALGSGIF. Analysis of its structure and physicochemical properties indicated that CBP22 had a molecular weight of 2264.63 Da and a +1 net charge. CBP22 showed activity against *E. col K88*, *E. coli ATCC25922*, and *S. aureus ATCC26923*. The effects and potential mechanisms of bacteriocin CBP22 on the innate immune response were investigated with a lipopolysaccharide- (LPS-) induced mouse model. The results showed that pretreatment with CBP22 prevented LPS-induced impairment in epithelial tissues and significantly reduced serum levels of IgG, IgA, IgM, TNF-*α*, and sIgA. Moreover, CBP22 treatment increased the expression of the zonula occludens and reduced permeability as well as apoptosis in the jejunum in LPS-treated mice. In summary, CBP22 inhibits the intestinal injury and prevents the gut barrier dysfunction induced by LPS, suggesting the potential use of CBP22 for treating intestinal damage.

## 1. Introduction

Prebiotics, first identified in 1905, have been used to manipulate microorganisms in the host to improve measurable health outcomes [1]. The probiotic *Clostridium butyricum* (*C. butyricum*) has been widely reported to have beneficial effects on hosts, including the prevention of bacterial and viral infections, immunoregulation, alleviation of acute pancreatitis and liver damage, and the reduction of lipogenesis, as well as having antitumor effects. We previously isolated the *C. butyricum* ZJU-F1 strain from the feces of a healthy pig and preserved it in the China General Microbiological Culture Collection Centre (CGMCC No. 8939, Shanghai, China). Our previous studies have shown that *C. butyricum* ZJU-F1 can alleviate growth retardation and intestinal barrier dysfunction caused by weaning [2]. However, the specific components of *C. butyricum* that are responsible for the beneficial biological functions are, as yet, unclear.

Much of our knowledge of the mechanisms of probiotics is based on bacteriocins. Bacteriocins are a class of bioactive polypeptides or proteins that are encoded by specific bacterial genes and synthesized in the ribosome. During the last decade, bacteriocins have been widely studied in the field of food preservation and alternative antibiotics. A growing number of studies have shown that bacteriocins have multiple biological functions, including antiviral activities, antiinfection of the gastrointestinal tract, spermicidal activities, and anticancer activity [3–6]. For example, plantaricin, a bacteriocin secreted by *Lactobacillus plantarum*, potentially benefits diet-induced obese mice via fortifying tight junctions between intestinal epithelial cells [7]. Bacteriocin Lmo2776 of *Listeria monocytogenes* can target the commensal *Prevotella copri* and modulate intestinal infection [8].

To date, three bacteriocins of *C. butyricum*, butyricin 7423, butyricum M588, and perfringocin 1105, with molecular weights of 32.5 kDa, 8 kDa, and 76.0 kDa, respectively, have been characterized [9, 10]. Sequence analysis of a bacteriocinogenic plasmid of *C. butyricum* found that butyricum M588 with 83 amino acids is encoded by the gene ORF3 and has high antibacterial activity against *Clostridium beijerinckii* and *C. pasteurianum* [10]. Butyricin 7423 alters the permeability of *C. pasteurianum* cell membranes, allowing the release of metabolites and ions [11]. It has been reported that the growth performance improvement of *C. butyricum* was associated with reducing or preventing the barrier damage caused by ETEC K88 in piglets and broiler chickens [12–14]. Mechanistically, studies have shown that *C. butyricum* regulation of immune response is through the TLR2-mediated MyD88-independent signaling pathway [15, 16]. However, the potential biological functions and mechanisms of action of bacteriocins secreted by *C. butyricum* in intestinal health have not yet been elucidated.

The intestine contains the largest number of immune cells of any tissue in the body to maintain tolerance and promote immune responses against a variety of clinically important pathogens [17, 18]. Irritable bowel syndrome (IBS), characterized by irregular bowel habits, affects 10% of the world's population in the 21^st^ century, but its aetiology remains largely undetermined [19, 20]. Studies have shown that intestinal infection, inflammation, and imbalance of intestinal microbiota can lead to IBS [21–23]. Several recent studies have shown a lower microbial diversity and a higher instability in the intestines of patients with IBS [20, 22]. Moreover, studies reported an increase in relative abundance of *Firmicutes*, mainly *Clostridium* cluster XIVa and *Ruminococcaceae*, together with a reduction in the relative abundance of *Bacteroidetes* in the intestines of patients with IBS [22, 24–26]. Our previous study suggested that maintaining tight junction complexes could modulate the inflammatory response to protect intestinal health [27]. Intestinal dysfunction plays a crucial role in the occurrence of systemic inflammation, suggesting that the intestine is the “engine” of systemic inflammation [28]. The intestinal mucosa forms a physical metabolic barrier, restricting the spread of pathogens, toxins, and allergens from the intestinal lumen to the circulatory system. The integrity of the intestinal barrier requires an accurate and delicate balance between proliferation and apoptosis [29, 30], the expression of tight junction proteins, such as ZO-1, ZO-2, occludin, and claudin-1 [31], and signaling molecules such as mucins, cytokines, Toll-like receptors, and chemokines [32].

In this study, we isolated and characterized the bacteriocin CBP22 from *Clostridium butyricum* ZJU-F1. The effects and potential mechanisms of bacteriocin CBP22 on the intestinal immune response were investigated by assessing alterations in the intestinal barrier with a lipopolysaccharide- (LPS-) induced intestinal injury mouse model. Taken together, we identified a new bacteriocin CBP22 and present evidence of its functions and mechanisms of action in immunomodulation.

## 2. Materials and Methods

### 2.1. Reagents

*Clostridium butyricum* ZJU-F1 came from our laboratory. *Staphylococcus aureus ATCC5923*, *Escherichia coli ATCC25922*, *Escherichia coli K88*, *Escherichia coli K12*, *Pseudomonas aeruginosa CMCC27853*, *Enterococcus faecalis EC533*, and *Enterococcus faecalis EC618* were purchased from the China General Microbiological Culture Collection Center. RCM media and Mueller-Hinton (MH) broth were purchased from Hopebio (Qingdao, China). Lipopolysaccharide (LPS) O111:B4 and 4 kDa fluorescein isothiocyanate-conjugated dextran (FD4) were purchased from Sigma-Aldrich.

### 2.2. Bacteriocin Purification

After culturing ZJU-F1 in RCM medium at 37°C and 225 rpm until the OD_600_ = 0.8, the supernatant was isolated. Tris-HCl (20 mM, pH = 8.0) was slowly added to the supernatant, followed by a very slow addition of ammonium sulfate at 4°C. After stirring for 2 h, the precipitate was collected by centrifugation (15,000 rpm, 10 min) and resuspended in binding buffer (20 mM Tris-HCl, 500 mM NaCl, 20 mM imidazole, 10 mM 2-mercaptoethanol, pH 8.0) and then dialyzed against the same buffer at 4°C for 12 h. Using the method of Luan et al. [33], the dialyzed preliminary extract was applied to a cation exchange column (GE Healthcare, Chicago, IL, USA) and was eluted by application of a salt gradient from 0 to 50% (*w*/*v*) NaCl in 20 mM Tris-HCl and 10 mM 2-mercaptoethanol (pH 8.0). All peaks were pooled and analyzed by sodium dodecyl sulfate-polyacrylamide gel electrophoresis (SDS-PAGE) and then dialyzed in binding buffer at 4°C for 12 h. The dialyzed mixture was then applied to a Ni-NTA resin column (1 ml) equilibrated with binding buffer, followed by a step gradient with elution buffer. Peaks were again analyzed by SDS-PAGE and collected separately before concentrating to 10 mg/ml. Each fraction was tested for antibacterial activity, and the fractions with antibacterial activity were further separated by reverse-phase high-performance liquid chromatography (RP-HPLC) [34]. The samples were dissolved in solvent A (5% acetonitrile containing 0.1% (*v*/*v*) TFA) and then applied to RP-HPLC. The separation was performed using a linear gradient from 15% to 45% solvent B (0.1% (*v*/*v*) TFA in 95% acetonitrile) for 40 min at a flow rate of 2.0 ml/min, measured at 215 nm. In short, the principle of eluting proteins with Ni NTA column was that the Ni NTA column contains agarose microspheres. Under the action of agarose chelating medium, the microspheres chelate with Ni^2+^, and the chelated Ni^2+^ can interact with the imidazole rings on His, Cys, or Trp, so as to achieve protein separation. Then, the sequences of purified proteins were determined by the Edman degradation method. The bacteriocin was synthesized by chemical synthesis based on the amino acid sequence, which was used for subsequent experiments *in vitro* and *in vivo*.

### 2.3. Antimicrobial Assays

The minimal inhibitory concentrations (MICs) were determined according to a modified version of the National Committee for Clinical Laboratory Standards broth microdilution method [35]. Microorganisms to be tested were grown to log phase in MH broth and transferred to fresh MH broth to a final concentration of 1 × 10^5^ CFU/ml. The concentrations of bacteriocin were diluted by twofold serial dilutions to 2.5, 5, 10, 20, 40, 80, 160, 320, 640, 1280, and 2560 *μ*g/ml. Bacterial solutions (90 ml) and bacteriocin (10 *μ*l) of different concentrations were added into a 96-well plate. The positive and negative controls were 100 *μ*l bacterial solution and 100 *μ*l MH broth, respectively. The MICs were measured after incubation for 24 h at 37°C. Each experiment was performed in triplicate.

### 2.4. Analysis of Structure and Physicochemical Properties

The Edman degradation method was used to determine the N-terminal amino acid sequence of the purified peptide according to the instructions of Applied Biosystems Company's gas-phase protein sequencer. The helical wheel plots, secondary structures, and physicochemical properties were predicted and analyzed by the online resources HeliQuest (https://heliquest.ipmc.cnrs.fr/) [36] and the Zhang laboratory at the University of Michigan (https://zhanglab.ccmb.med.umich.edu/) [37].

### 2.5. Hemolysis Assay

The hemolysis test was carried out using the method described by Gao et al. [35]. Briefly, fresh anticoagulated pig blood was collected from a healthy donor (Hangzhou, China) in a polycarbonate tube containing heparin. The blood was washed twice with PBS and then diluted to 1% in PBS with or without 10% fetal bovine serum. CBP22 was serially diluted with 0.01% acetic acid to 0 *μ*g/ml to 256 *μ*g/ml. Different concentrations (10 *μ*l) of CBP22 were incubated with erythrocytes (90 *μ*l) at 37°C for 2 h in a 96-well plate. After centrifugation, the supernatants were transferred to a new 96-well plate to measure the absorbance at 405 nm. The negative and positive controls were erythrocytes incubated in 10 *μ*l 0.01% acetic acid and 1% Triton X-100, respectively. Hemolysis (%) was calculated as follows:

(1) $$\begin{array}{c} \text{Hemolysis}\left(\%\right)=\frac{A_{\text{antimicrobial peptide}}-A_{0.01\%\text{acetic acid}}}{A_{1\%\text{Triton X}-100}-A_{0.01\%\text{acetic acid}}}*100. \end{array}$$

### 2.6. Animals and Experimental Design

Sixty ICR male mice, aged 4-6 weeks, purchased from Shanghai SLAC Laboratory Animal Central, were used. The mice were raised in plastic cages under standard conditions (12 h light-dark cycle, 22-25°C, humidity 50-70%) and with free access to food and water. All animal experiments were carried out under the Animal Care and Use Committee of Zhejiang University.

**Figure 1:**
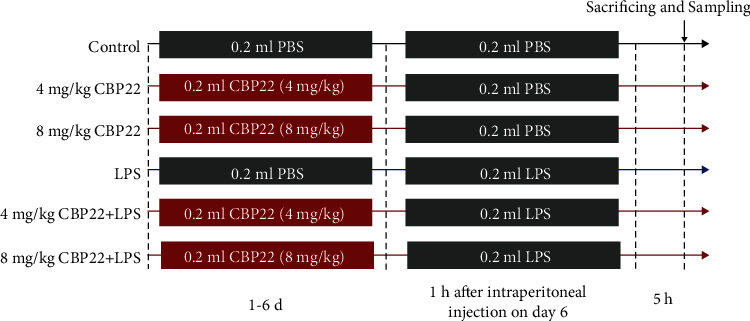
Experimental design and scheme of animal treatments.

As shown in Figure 1, the mice were randomly divided into six groups of 10 each. On day 6, mice in the LPS and CBP22+LPS groups were intraperitoneally injected with LPS (10 mg/kg, 200 *μ*l each mouse) 1 h after CBP22 or PBS treatment; the other groups were injected with an equal volume of PBS. The mice were sacrificed, and blood samples were collected by cardiac puncture 5 h after LPS stimulation. In addition, both LPS and CBP22 were dissolved in PBS before administration.

### 2.7. Intestinal Histomorphology

The jejunum samples of mice were fixed in 4% paraformaldehyde for 24 h and then embedded in paraffin blocks. Sections of 5 *μ*m thickness were cut and stained with hematoxylin and eosin (H&E), and images were acquired through a Leica DM3000 Microsystem (Leica, Germany). The villus height and crypt depth were measured using a Leica microscope (DM3000; Leica, Wetzlar, Germany) equipped with a CCD camera (DFC420; Leica). All programs were executed three times, and the data presented is the average of the three replicates. The degree of small intestinal injury was evaluated by Chiu's score classification [38] as follows: grade zero, normal mucosal villi; grade one, well-structured villi but subepithelial spaces; grade two, elevated villous epithelium with increasing subepithelial spaces; grade three, some cast-off villous epithelium; grade four, structural destruction of villi resulting in shedding and telangiectasia; and grade five, destruction of all the mucosa, hemorrhage, and ulceration.

### 2.8. Intestinal Permeability

The assessment of intestinal permeability is based on the measurement of FD4 in serum. Mice that had been intraperitoneally injected with LPS or PBS were given FD4 by oral gavage (20 mg/kg body weight) after 1 h. The blood collected at the time of sacrifice was centrifuged at 12,000 rpm at 4°C for 5 minutes. The concentration of FD4 in the serum was determined by measuring fluorescence using a SpectraMax M5 plate reader (Molecular Devices, San Jose, CA, USA) with an excitation wavelength of 485 nm and an emission wavelength of 535 nm.

### 2.9. TUNEL Staining

The TUNEL assay was used to identify jejunal epithelial cell apoptosis. The Leica DM3000 microsystem was used to analyze labeled cells. At least five views of each image from the different groups were taken with the background light kept constant between images. Five random duplications from each group were analyzed, the ratio of apoptotic to nonapoptotic cells was calculated according to the positive brown coloration, and the average values were calculated. The apoptosis index (AI) was calculated according to the following formula:

(2) $$\begin{array}{c} \text{AI}=\frac{\text{ the number of apoptosis cells }\left(\text{AC}\right)}{\text{AC}+\text{the number of intact cells }\left(\text{IC}\right)}*100\%. \end{array}$$

### 2.10. Measurement of Immunoglobulins and TNF-*α* in Serum

The serum levels of immunoglobulins (IgG, IgA, and IgM) and TNF-*α* were determined using an enzyme-linked immunosorbent assay (ELISA) kit (Multisciences, Hangzhou, China) according to the manufacturer's protocol.

### 2.11. Determination of Intestinal sIgA Concentrations

The expression level of sIgA in ileal tissue was determined by a double-antibody sandwich ELISA, as previously described [39] using an ELISA kit purchased from Multisciences (Hangzhou, China). The ileal protein concentrations were measured by the BCA kit (KeyGEN, China), and the concentrations of sIgA were expressed as *μ*g sIgA/mg protein.

### 2.12. Real-Time PCR

Total RNA was extracted from the jejunum using the TRIzol reagent according to the manufacturer's instructions. The RNA was reverse-transcribed into cDNA using a cDNA Reverse Transcription Kit. The sequences of the gene-specific primers were determined from previous publications [40] and were synthesized and purchased from Sangon Biotech (Shanghai, China). The primer sequences and their Tm values and sizes are listed in Table 1. Amplification and detection were carried out using the SYBR Premix Ex Taq Kit (Takara Clontech, Otsu, Japan) in the ABI StepOnePlus Real-time PCR system (Applied Biosystems, Foster City, CA, USA). All the samples were analyzed in triplicate, and negative controls were included to check for the nonspecific amplification of primers.

### 2.13. Statistical Analysis

All statistical analyses were performed using SPSS software (Version 20.0, IBM Corp., Armonk, NY, USA), and all data were expressed as the means ± standard error (SEM). Significant differences between the control and experimental groups were determined by a one-way ANOVA with Duncan's multiple range test. Statistically, a *P* value of less than 0.05 was considered significant.

## 3. Results

### 3.1. Isolation, Purification, and Identification of CBP22

**Figure 2:**
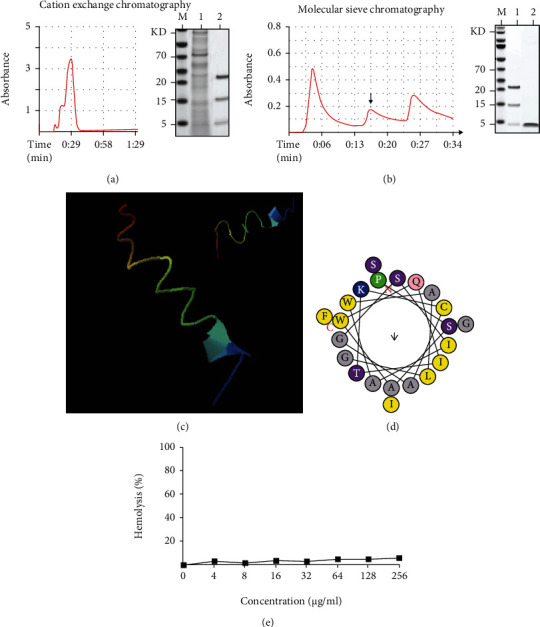
Isolation and identification of CBP22. (a) Cation exchange chromatography of the supernatant from *C. butyricum* culture medium at a wavelength of 215 nm and SDS-PAGE analysis. M: mark; lane 1: before purification; lane 2: after purification. (b) Affinity chromatography of products from (a) at a wavelength of 215 nm and SDS-PAGE analysis. M: mark; lane 1: before purification; lane 2: after purification. (c) Secondary structure prediction of CBP22. (d) Helical wheel plots of CBP22. (e) Hemolytic activity of CBP22 against porcine red blood cells. The hemoglobin release was monitored at 576 nm. The data are expressed as the mean ± SEM, *n* = 3 biological replicates; bars with different small capital letters are statistically different from one another.

The bacteriocin in the supernatant of ZJU-F1 was preliminarily extracted by ammonium sulfate precipitation and then separated and purified by cation exchange column chromatography, yielding three fractions (Figure 2(a)). The three fractions were separated and collected by affinity chromatography (Figure 2(b)). We then tested the antibacterial activity of the three fractions and found that only the second fraction showed antibacterial activity against *E. coil* and *S. aureus.* Thus, the second fraction was freeze-dried and concentrated, followed by purification on RP-HPLC. SDS-PAGE analysis showed that the molecular weight of the second fraction was about 3 kDa. Sequencing showed that the bacteriocin was a 22-amino acid peptide with the sequence PSAWQITKCAGSIAWALGSGIF; the peptide was, therefore, named *Clostridium butyricum* peptide 22 (CBP22).

To further investigate the physicochemical properties of CBP22, we used helical wheel plots and secondary structure prediction by online analysis tools (Figures 2(c) and 2(d)). The results indicated that the isoelectric point of CBP22 was 8.64 and the molecular weight was 2264.64 Da, with a net charge of +1 and hydrophobicity of 0.719 and a hydrophobic moment of 0.134.

### 3.2. Antibacterial Activity of CBP22

To evaluate the antibacterial activity of CBP22, the minimal inhibitory concentration (MIC) of the bacteriocin CBP22 against pathogenic bacteria was determined. As Table 2 shows, the CBP22 exhibited moderate activity against *E. coli K88* with a MIC of 32 *μ*g/ml and showed low activity against *E. coli ATCC25922* and *S. aureus ATCC26923*, with MICs of 128 and 64 *μ*g/ml, respectively.

### 3.3. Hemolytic Activity

The hemolytic activity of the CBP22 against human erythrocytes was determined as a measure of toxicity to mammalian cells. The results showed that CBP22 had a low lethality to erythrocytes between 0 and 256 *μ*g/ml. Fewer than 10% of erythrocytes were killed even at 256 mg/ml (Figure 2(e)). These results indicated that CBP22 is safe to eukaryotes.

### 3.4. Intestinal Morphology

**Figure 3:**
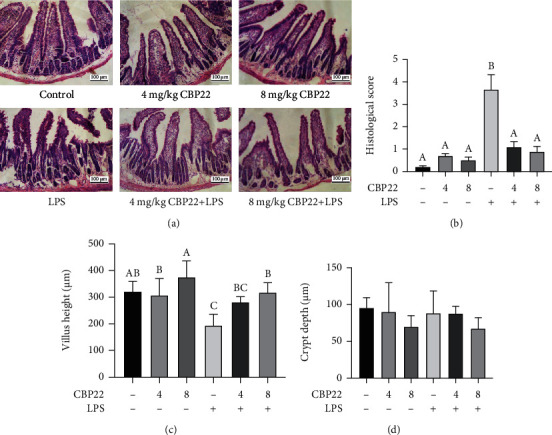
The protective effects of CBP22 on jejunal morphology in mice treated with LPS. (a) H&E staining of the jejunal mucosa, scale bar = 100 *μ*m. (b) Mucosal damage grading. (c) Villus heights in the jejunum. (d) Crypt depths in the jejunum. The data are expressed as the mean ± SEM, *n* = 10 biological replicates; bars with different small capital letters are statistically different from one another.

To further investigate the effects of CBP22 on intestinal health, we induced a mouse model with LPS stimulation. As shown in Figure 3(a), H&E staining of jejunal specimens from the control and CBP22-treated groups showed integrated villi and compactly arrayed epithelium. On the contrary, the intestinal epithelia of the jejunum in LPS-treated mice showed marked injury with epithelial exfoliation, discontinuous brush borders, and blunt villi. However, the jejunal villi of mice pretreated with 4 mg/kg and 8 mg/kg CBP22 were relatively intact, which suggested that CBP22 significantly alleviated the injury caused by LPS to the villi. Based on the score of small intestinal mucosal injury, we found that pretreatment with CBP22 prevented intestinal mucosal injury induced by LPS (Figure 3(b)).

As Figures 3(c) and 3(d) show, compared with the control group, the jejunal villus height and crypt depth in mice treated with 4 mg/kg and 8 mg/kg CBP22 were not significantly different while high concentrations of CBP22 caused a trend of increasing villus height and decreasing crypt depth. However, the villus height in the LPS group was reduced without significant alteration of the crypt depth. CBP22 pretreatment significantly alleviated the villus shortening caused by LPS with an improvement of 27.6% after treatment with 8 mg/kg CBP22 but with no effect on the crypt depth. These results suggest that CBP22 can protect the intestinal villi of mice from LPS damage.

### 3.5. Intestinal Epithelial Cell Apoptosis

**Figure 4:**
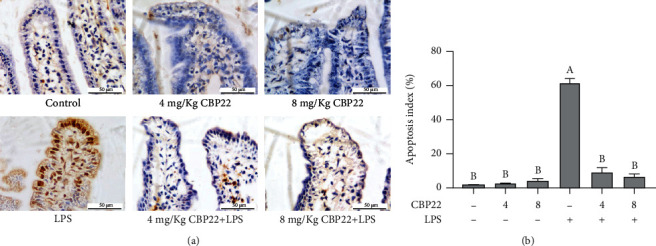
The effect of CBP22 on jejunum cell apoptosis in mice. (a) TUNEL staining of jejunal epithelial tissues, brown signals, scale bar = 50 *μ*m. (b) Apoptosis index of jejunal epithelial cells. The data are expressed as the mean ± SEM, *n* = 10 biological replicates; bars with different small capital letters are statistically different from one another.

Tissue TUNEL staining was conducted to analyze the levels of apoptosis in the jejunum. The brown and blue staining represented the apoptotic and normal nuclei, respectively. In contrast to the control group, the apoptosis index of the 4 mg/kg and 8 mg/kg CBP22 treatments showed no significant difference, but LPS-treated mice showed a significantly increased jejunal apoptosis index. Compared with LPS-treated mice, in the mice pretreated with 4 mg/kg and 8 mg/kg CBP22 followed by treatment with LPS, the apoptosis index was significantly decreased by 82.65% and 86.81%, respectively (Figures 4(a) and 4(b)).

### 3.6. Serum Levels of IgA, IgG, IgM, and TNF-*α*

**Figure 5:**
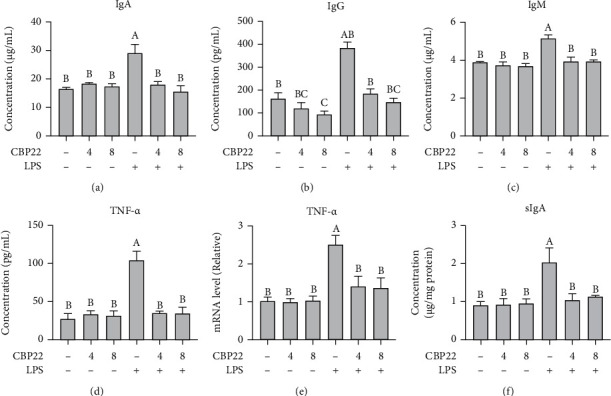
Effects of CBP22 on immune function in LPS-stimulated mice. (a–d) The concentration of IgA (a), IgG (b), IgM (c), and TNF-*α* (d) in serum. (e) Real-time PCR analysis of TNF-*α* mRNA expression in the jejunum. (f) Concentration of sIgA in the ileum. The q-PCR results are presented relative to those of GAPDH. The data are expressed as the mean ± SEM, *n* = 10 biological replicates; bars with different small capital letters are statistically different from one another.

To determine the effects of CBP22 on the LPS-induced immune response, the levels of immunoglobulins and cytokines were measured. LPS treatment resulted in a marked increase in serum levels of IgA, IgG, and IgM compared with the control. CBP22 pretreatment significantly alleviated the elevation of serum immunoglobulins induced by LPS (Figures 5(a)–5(c)).

As shown in Figure 5(d), the serum TNF-*α* concentration of mice in the 4 mg/kg and 8 mg/kg CBP22 groups was slightly elevated but not significantly so. LPS treatment resulted in a 3.85-fold increase in serum TNF-*α* concentration relative to the control group (104.31 ± 11.63 pg/ml vs. 27.10 ± 7.14 pg/ml). CBP22 pretreatment significantly reduced TNF-*α* secretion induced by LPS to the same level as the control group.

### 3.7. Intestinal sIgA and TNF-*α* Level

To further confirm the effects of CBP22 on the immune response, the TNF-*α* mRNA expression in the jejunum was determined. Similar to the results in the serum, there was no significant difference between TNF-*α* mRNA in the jejunum of mice treated with 4 mg/kg and 8 mg/kg CBP22. The expression of TNF-*α* mRNA in the jejunum of LPS-treated mice was significantly increased, but pretreatment with 4 mg/kg and 8 mg/kg CBP22 followed by LPS resulted in significantly lower levels than those of the LPS group (Figure 5(e)).

The results of the ileal sIgA levels are shown in Figure 5(f). In comparison with the control mice, there was no significant difference in the level of sIgA in the terminal ileum of mice treated with 4 mg/kg and 8 mg/kg CBP22. However, LPS treatment resulted in a 123.08% increase in the sIgA level relative to the control group. Pretreatment with 4 mg/kg and 8 mg/kg CBP22 significantly decreased secretion of sIgA by 40.39% and 43.84%, respectively, with no difference from the control group.

### 3.8. Intestinal Permeability

**Figure 6:**
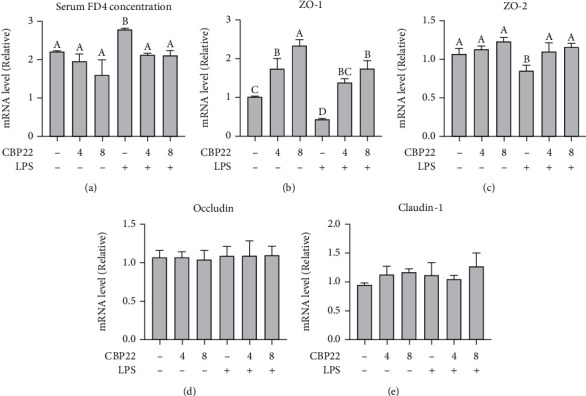
The protective effects of CBP22 on the intestinal barrier. (a) Intestinal permeability is determined by serum FD4 concentration. (b–d) RT-PCR analysis. The mRNA expression of tight junctions and results are presented relative to those of GAPDH, (b) ZO-1, (c) ZO-2, (d) occludin, and (e) claudin-1. The data are expressed as the mean ± SEM, *n* = 10 biological replicates; bars with different small capital letters are statistically different from one another.

As the gut barrier function can regulate immune function, we assessed the intestinal permeability in each group. As shown in Figure 6(a), after intraperitoneal injection with LPS, the intestinal permeability to FD4 was significantly increased indicating that the barrier function was impaired. Pretreatment of LPS-administered mice with 4 mg/kg and 8 mg/kg CBP22 significantly reduced the FD4 concentration in the serum indicating that CBP22 can protect the mouse intestinal barrier function against LPS challenge. However, there was no significant difference between CBP22 treatment alone and the control groups.

### 3.9. Intestinal Tight Junction Expression

To further investigate the role of CBP22 in intestinal barrier function, tight junction markers, such as claudin-1, occludin, ZO-1, and ZO-2, were measured. The mRNA expression levels of ZO-1 in the jejunum of mice treated with 4 mg/kg and 8 mg/kg CBP22 alone increased significantly in a dose-dependent manner (Figure 6(b)). LPS markedly reduced the mRNA expression of ZO-1 and ZO-2 by 57% and 15%, respectively, but did not affect claudin-1 and occludin expression compared with the control group. As expected, CBP22 pretreatment completely and significantly abrogated the LPS-induced reduction in ZO-1 and ZO-2 mRNA (Figures 6(b)–6(d)).

## 4. Discussion

In this study, we describe the isolation of a novel bacteriocin from *C. butyricum* ZJU-F1 named CBP22 with a molecular weight of 2264.63 Da. We then demonstrated the role and potential mechanism of action of CBP22 effect on intestinal injury using an LPS-induced mouse model.

In recent years, studies found that many bacteriocins, such as SA2715 [41], MQ1 [42], and Nisin [43], have great potential as natural preservatives in food. In addition, bacteriocin is also widely used in oral care, skincare, vaginal care, and anticancer drugs [43–46]. In contrast to the other three known bacteriocins of *C. butyricum*, the molecular weight of CBP22 was relatively small, only 2264.35 Da, which is conducive to industrial production and a wide application in preservatives in the food and the care of oral, skin, and vaginal [43, 47]. In general, bacteriocins have a high toxicity. Hemolysis is an important indicator to evaluate the safety of bacteriocin. If the hemolysis rate is less than 5%, the bacteriocin is considered to have no hemolysis [48]. Our results showed that CBP22 had a very low hemolytic activity with less than 5%, indicating its potential safety. Moreover, our results found that CBP22 showed antibacterial activity against *E. coli* and *S. aureus* while not affecting *P. aeruginosa*, *E. faecium*, or *E. faecalis*, indicating that CBP22 may be a bacteriocin with narrow-spectrum antibacterial activity. Meanwhile, the physicochemical properties of CBP22 showed that it had one positive charge, which may be the reason for its weak antibacterial activity [49]. If CBP22 is used as the model to design derivative peptides, by changing one or more amino acids to increase the charge number and hydrophobic moment, a derivative peptide with high antibacterial activity and narrower antibacterial spectrum can be found, which will be conducive to the development of antibacterial specific and efficient bacteriocin.

In addition to antibacterial activity, the immune regulatory function of bacteriocins has attracted increasing attention. LPS is commonly used to induce acute immune responses in mammals, destroying the self-renewal of the intestinal villi and resulting in a high cell apoptosis index. The intestinal mucosa is the major site of nutrient absorption and digestion [50]. Intestinal diseases can directly destroy the ability to absorb and digest nutrients and may even cause inflammatory-related diseases that affect the whole body. It has been reported that bacteriocins and antimicrobial peptide can regulate tissue half-life and clearance of neutrophils by suppressing apoptosis [51]. Our results have shown that CBP22 alleviated intestinal damage caused by LPS and confirmed marked injury and a high cell apoptosis index for the jejunal intestinal epithelia of LPS-treated mice using H&E staining. As expected, all of these effects were significantly inhibited by CBP22 injection.

Bacteriocins modulate the immune response through regulating inflammation, inducing chemotaxis, initiating specific immunity, directly enhancing the ability of the body to fight infection, and specifically activating immune cells [4, 44]. About bacteriocin regulation of intestinal immunity, the study found that a *Listeria monocytogenes* bacteriocin could target the commensal *Prevotella copri* and modulate intestinal infection [8]. Besides, it has also been found that gassericin A, a bacteriocin, secreted by intestinal microorganisms, bound KRT19 protein to the plasma membrane of intestinal epithelial cell, and activates intracellular mTOR-mediated phosphodiesterase activity, resulting in the downregulation of cAMP and cGMP levels, thereby reducing the incidence of diarrhea in early-weaned piglets [52]. IgG, IgM, and IgA, important effector molecules in humoral immunity, can neutralize the toxicity of bacterial toxins, enhance the phagocytosis of mononuclear macrophages, and bind viral antigens to regulate immunity [53, 54]. Consistent with previous studies [55], our data showed that LPS significantly increased the levels of IgA, IgG, and IgM in mouse serum. Although CBP22 treatment alone did not affect the serum immunoglobulin levels, pretreatment with CBP22 could reduce the elevated immunoglobulin levels induced by LPS. These results suggested that CBP22 can activate the nonspecific immunity of mice to neutralize and eliminate LPS. sIgA, a major component of the mucosal defense system, can inhibit the adhesion of intestinal epithelial pathogenic microorganisms and the proliferation of viruses. Our results showed that sIgA concentration was dramatically increased after intraperitoneal LPS injection but pretreatment with CBP22 decreased the sIgA concentration in the ileum, indicating that CBP22 may inhibit this extreme immune response to protect the health of the mouse. Besides, after intraperitoneal LPS injection, the serum levels of TNF-*α* and the gene expression of TNF-*α* in the small intestine increased rapidly, possibly because LPS activated inflammatory signaling pathways such as NF-*κ*B and MAPK, thereby activating the expression of downstream proinflammatory factors [56]. Our results suggested that pretreatment with CBP22 can significantly reduce the TNF-*α* level in serum and the TNF-*α* mRNA level in the small intestine, which may be because CBP22 alleviates inflammation by inhibiting I*κ*B degradation and NF-*κ*B activation [57].

The pathogenesis of sepsis has been attributed, at least in part, to the loss of intestinal epithelial barrier [58]. As the first line of defense, the barrier plays a vital role in maintaining intestinal health, preventing the uptake of pathogenic microorganisms, bacterial endotoxins, antigens, and toxic macromolecules from the lumen to the gut [59]. *Lactobacillus plantarum* bacteriocin improved the gut and body of diet-induced obese mice by maintaining the integrity of the epithelial barrier [7]. Mechanistically, bacteriocin secreted by probiotic lactobacilli was involved in the maintenance of the mucosal barrier, mainly through MAPK-dependent mechanisms [60]. To investigate the potential mechanism of CBP22 on immune function, we evaluated the effect of CBP22 on intestinal epithelial barrier function in mice. Healthy intestinal tissue was less permeable to FD4 to the blood, which was released into the blood only when intestinal mucosal barrier was damaged, so their levels could directly reflect the degree of damage to the intestinal epithelial mucosa and permeability [61]. We found that CBP22 reduced the level of FD4, indicating that CBP22 alleviated LPS-induced permeability. Tight junction proteins, such as ZO-1, ZO-2, occludin, and claudin-1, are important factors regulating intestinal permeability and are the major barrier components. Our results showed that LPS downregulated the gene expression of the tight junction proteins ZO-1 and ZO-2 resulting in deterioration of the gut structure. These results further confirmed the conclusion that CBP22 may regulate intestinal injury by maintaining the barrier function.

In conclusion, we isolated and characterized a novel bacteriocin CBP22 from *C. butyricum.* Based on the analysis of its structure, physicochemical properties, antibacterial activity, and safety, we present evidence supporting the role of CBP22 as a potential regulator of intestinal injury and explore its mechanisms of action through enhancing the intestinal barrier function.

## Figures and Tables

**Table 1:** Specific primers used for real-time PCR.

Primer	Sequence (5′–3′)	Length (bp)		Tm (°C)
ZO-1	F	TCATCCCAAATAAGAACAGAGC	198	60
	R	GAAGAACAACCCTTTCATAAGC		

ZO-2	F	GCTTTGGTGTGGACCAAGAT	269	60
	R	TCCATTATGGGTTTGCATGA		

Claudin-1	F	GCTGGGTTTCATCCTGGCTTCT	110	60
	R	CCTGAGCGGTCACGATGTTGTC		

Occludin	F	AAGCAAGTGAAGGGATCTGC	204	60
	R	GGGGTTATGGTCCAAAGTCA		
TNF-*α*	F	GCATGGTGGTGGTTGTTTCTGACGAT	99	60
	R	GCTTCTGTTGGACACCTGGAGACA		

GAPDH	F	CAACGGCACAGTCAAGGCTGAGA	112	60
	R	CTCAGCACCAGCATCACCCCAT		

**Table 2:** Antibacterial activity of CBP22 measured by MIC.

Bacterial species	MIC (*μ*g/ml)
Gram-negative bacteria	
*E. col K88*	32
*E. col ATCC25922*	128
*E. col K12*	>256
*P. aeruginosa CMCC27853*	>256
Gram-positive bacteria	
*S. aureus ATCC26923*	64
*E. faecium EC533*	>256
*E. faecalis EC618*	>256

## Data Availability

The data of qRT-PCR, Western blot, and ELISA used to support the findings of this study are available from the corresponding authors upon request.

## References

[1] Abraham B. P., Quigley E. M. M. (2017). Probiotics in inflammatory bowel disease. *Gastroenterology Clinics of North America*.

[2] Zong X., Wang T. H., Lu Z. Q., Song D. G., Zhao J., Wang Y. Z. (2019). Effects of *Clostridium butyricum* or in combination with _Bacillus licheniformis_ on the growth performance, blood indexes, and intestinal barrier function of weanling piglets. *Livestock Science*.

[3] Quintana V. M., Torres N. I., Wachsman M. B., Sinko P. J., Castilla V., Chikindas M. (2014). Antiherpes simplex virus type 2 activity of the antimicrobial peptide subtilosin. *Journal of Applied Microbiology*.

[4] Quereda J. J., Dussurget O., Nahori M.-A. (2016). Bacteriocin from epidemic Listeria strains alters the host intestinal microbiota to favor infection. *Proceedings of the National Academy of Sciences*.

[5] Sutyak K. E., Anderson R. A., Dover S. E. (2008). Spermicidal activity of the safe natural antimicrobial peptide subtilosin. *Infectious Diseases in Obstetrics and Gynecology*.

[6] Ahmadi S., Ghollasi M., Hosseini H. M. (2017). The apoptotic impact of nisin as a potent bacteriocin on the colon cancer cells. *Microbial Pathogenesis*.

[7] Heeney D. D., Zhai Z., Bendiks Z. (2019). Lactobacillus plantarumbacteriocin is associated with intestinal and systemic improvements in diet-induced obese mice and maintains epithelial barrier integrityin vitro. *Gut Microbes*.

[8] Rolhion N., Chassaing B., Nahori M.-A. (2019). A *Listeria monocytogenes* Bacteriocin Can Target the Commensal _Prevotella copri_ and Modulate Intestinal Infection. *Cell Host Microbe*.

[9] Clarke D. J., Robson R. M., Morris J. G. (1975). Purification of two Clostridium bacteriocins by procedures appropriate to hydrophobic proteins. *Antimicrob Agents Chemother*.

[10] Nakanishi S., Tanaka M. (2010). Sequence analysis of a bacteriocinogenic plasmid of Clostridium butyricum and expression of the bacteriocin gene in Escherichia coli. *Anaerobe*.

[11] Clarke D. J., Morley C. D., Kell D. B., Morris J. G. (1982). On the mode of action of the bacteriocin butyricin 7423. Effects on membrane potential and potassium-ion accumulation in Clostridium pasteurianum. *European Journal of Biochemistry*.

[12] Li H. H., Li Y. P., Zhu Q., Qiao J. Y., Wang W. J. (2018). Dietary supplementation with Clostridium butyricum helps to improve the intestinal barrier function of weaned piglets challenged with enterotoxigenic Escherichia coli K88. *Journal of Applied Microbiology*.

[13] Fu J., Wang T., Xiao X. (2021). Clostridium butyricum ZJU-F1 benefits the intestinal barrier function and immune response associated with its modulation of gut microbiota in weaned piglets. *Cells*.

[14] Zhang L., Zhang L., Zhan X. (2016). Effects of dietary supplementation of probiotic, Clostridium butyricum, on growth performance, immune response, intestinal barrier function, and digestive enzyme activity in broiler chickens challenged with Escherichia coli K88. *Journal of Animal Science and Biotechnology*.

[15] Gao Q., Qi L., Wu T., Wang J. (2012). Clostridium butyricum activates TLR2-mediated MyD88-independent signaling pathway in HT-29 cells. *Molecular and Cellular Biochemistry*.

[16] Sui S. J., Tian Z. B., Wang Q. C. (2018). Clostridium butyricum promotes intestinal motility by regulation of TLR2 in interstitial cells of Cajal. *European Review for Medical and Pharmacological Sciences*.

[17] Mowat A. M., Agace W. W. (2014). Regional specialization within the intestinal immune system. *Nature Reviews Immunology*.

[18] Zong X., Fu J., Xu B., Wang Y., Jin M. (2020). Interplay between gut microbiota and antimicrobial peptides. *Animal Nutrition*.

[19] Zhao L., Yang W., Chen Y. (2020). A Clostridia-rich microbiota enhances bile acid excretion in diarrhea-predominant irritable bowel syndrome. *The Journal of Clinical Investigation*.

[20] Enck P., Aziz Q., Barbara G. (2016). Irritable bowel syndrome. *Nature Reviews Disease Primers*.

[21] Simrén M., Barbara G., Flint H. J. (2013). Intestinal microbiota in functional bowel disorders: a Rome foundation report. *Gut*.

[22] Rajilić-Stojanović M., Jonkers D. M., Salonen A. (2015). Intestinal microbiota and diet in IBS: causes, consequences, or epiphenomena?. *American Journal of Gastroenterology*.

[23] Bischoff S. C., Barbara G., Buurman W. (2014). Intestinal permeability--a new target for disease prevention and therapy. *BMC Gastroenterology*.

[24] Salonen A., de Vos W. M., Palva A. (2010). Gastrointestinal microbiota in irritable bowel syndrome: present state and perspectives. *Microbiology (Reading)*.

[25] Jeffery I. B., O'Toole P. W., Öhman L. (2012). An irritable bowel syndrome subtype defined by species-specific alterations in faecal microbiota. *Gut*.

[26] Krogius-Kurikka L., Lyra A., Malinen E. (2009). Microbial community analysis reveals high level phylogenetic alterations in the overall gastrointestinal microbiota of diarrhoea-predominant irritable bowel syndrome sufferers. *BMC Gastroenterology*.

[27] Zong X., Hu W., Song D. (2016). Porcine lactoferrin-derived peptide LFP-20 protects intestinal barrier by maintaining tight junction complex and modulating inflammatory response. *Biochemical Pharmacology*.

[28] Hassoun H. T., Kone B. C., Mercer D. W., Moody F. G., Weisbrodt N. W., Moore F. A. (2001). Post-injury multiple organ failure: the role of the gut. *Shock*.

[29] Hall P. A., Coates P. J., Ansari B., Hopwood D. (1994). Regulation of cell number in the mammalian gastrointestinal tract: the importance of apoptosis. *Journal of Cell Science*.

[30] Watson A. J. M., Chu S., Sieck L. (2005). Epithelial barrier function in vivo is sustained despite gaps in epithelial layers. *Gastroenterology*.

[31] Li Q., Zhang Q., Wang C., Liu X., Li N., Li J. (2009). Disruption of tight junctions during polymicrobial sepsis in vivo. *The Journal of Pathology*.

[32] Arce C., Ramírez-Boo M., Lucena C., Garrido J. J. (2010). Innate immune activation of swine intestinal epithelial cell lines (IPEC-J2 and IPI-2I) in response to LPS from Salmonella typhimurium. *Comparative Immunology, Microbiology and Infectious Diseases*.

[33] Luan C., Zhang H. W., Song D. G., Xie Y. G., Feng J., Wang Y. Z. (2014). Expressing antimicrobial peptide cathelicidin-BF in Bacillus subtilis using SUMO technology. *Applied Microbiology and Biotechnology*.

[34] Song R., Wei R.-B., Luo H.-Y., Wang D.-F. (2012). Isolation and characterization of an antibacterial peptide fraction from the pepsin hydrolysate of half-fin anchovy (Setipinna taty). *Molecules*.

[35] Gao W., Xing L., Qu P. (2015). Identification of a novel cathelicidin antimicrobial peptide from ducks and determination of its functional activity and antibacterial mechanism. *Scientific Reports*.

[36] Gautier R., Douguet D., Antonny B., Drin G. (2008). HELIQUEST: a web server to screen sequences with specific alpha-helical properties. *Bioinformatics*.

[37] Yang J., Yan R., Roy A., Xu D., Poisson J., Zhang Y. (2015). The I-TASSER Suite: protein structure and function prediction. *Nature Methods*.

[38] Chiu C. J., McArdle A. H., Brown R., Scott H. J., Gurd F. N. (1970). Intestinal mucosal lesion in low-flow states. *Archives of Surgery*.

[39] Snel J., Bakker M. H., Heidt P. J. (1997). Quantification of antigen-specific immunoglobulin A after oral booster immunization with ovalbumin in mice mono-associated with segmented filamentous bacteria or Clostridium innocuum. *Immunology Letters*.

[40] Zong X., Zhao J., Wang H. (2019). Mettl3 deficiency sustains long-chain fatty acid absorption through suppressing Traf6-dependent inflammation response. *Journal of Immunology*.

[41] Wayah S. B., Philip K. (2018). Characterization, yield optimization, scale up and biopreservative potential of fermencin SA715, a novel bacteriocin from Lactobacillus fermentum GA715 of goat milk origin. *Microbial Cell Factories*.

[42] Wayah S. B., Philip K. (2018). Pentocin MQ1: a novel, broad-spectrum, pore-forming bacteriocin from Lactobacillus pentosus CS2 with quorum sensing regulatory mechanism and biopreservative potential. *Frontiers in Microbiology*.

[43] Juturu V., Wu J. C. (2018). Microbial production of bacteriocins: latest research development and applications. *Biotechnology Advances*.

[44] Baindara P., Korpole S., Grover V. (2018). Bacteriocins: perspective for the development of novel anticancer drugs. *Applied Microbiology and Biotechnology*.

[45] Cotter P. D., Hill C., Ross R. P. (2005). Bacteriocins: developing innate immunity for food. *Nature Reviews Microbiology*.

[46] O’Connor P. M., Kuniyoshi T. M., Oliveira R. P. S., Hill C., Ross R. P., Cotter P. D. (2020). Antimicrobials for food and feed; a bacteriocin perspective. *Current Opinion in Biotechnology*.

[47] Kimura K., Yokoyama S. (2019). Trends in the application of Bacillus in fermented foods. *Current Opinion in Biotechnology*.

[48] Lopez F. E., Vincent P. A., Zenoff A. M., Salomón R. A., Farías R. N. (2007). Efficacy of microcin J25 in biomatrices and in a mouse model of Salmonella infection. *Journal of Antimicrobial Chemotherapy*.

[49] Zasloff M. (2002). Antimicrobial peptides of multicellular organisms. *Nature*.

[50] Gu L., Li N., Gong J., Li Q., Zhu W., Li J. (2011). Berberine ameliorates intestinal epithelial tight-junction damage and down-regulates myosin light chain kinase pathways in a mouse model of endotoxinemia. *The Journal of Infectious Diseases*.

[51] van der Does A. M., Hiemstra P. S., Mookherjee N. (2019). Antimicrobial host defence peptides: immunomodulatory functions and translational prospects. *Advances in Experimental Medicine and Biology*.

[52] Hu J., Ma L., Nie Y. (2018). A microbiota-derived bacteriocin targets the host to confer diarrhea resistance in early-weaned piglets. *Cell Host Microbe*.

[53] Macpherson A. J., Yilmaz B., Limenitakis J. P., Ganal-Vonarburg S. C. (2018). IgA function in relation to the intestinal microbiota. *Annual Review of Immunology*.

[54] Schroeder H. W., Cavacini L. (2010). Structure and function of immunoglobulins. *The Journal of Allergy and Clinical Immunology*.

[55] Li P., Yao Y., Ma Y., Chen Y. (2019). miR-150 attenuates LPS-induced acute lung injury via targeting AKT3. *International Immunopharmacology*.

[56] Copeland S., Warren H. S., Lowry S. F., Calvano S. E., Remick D. (2005). Acute inflammatory response to endotoxin in mice and humans. *Clinical Diagnostic Laboratory Immunology*.

[57] Gao Y., Lecker S., Post M. J. (2000). Inhibition of ubiquitin-proteasome pathway–mediated I*κ*B*α* degradation by a naturally occurring antibacterial peptide. *Journal of Clinical Investigation*.

[58] Ho J., Chan H., Liang Y. (2020). Cathelicidin preserves intestinal barrier function in polymicrobial sepsis. *Critical Care*.

[59] Turner J. R. (2009). Intestinal mucosal barrier function in health and disease. *Nature Reviews Immunology*.

[60] Dicks L. M. T., Dreyer L., Smith C., van Staden A. D. (2018). A review: the fate of bacteriocins in the human gastro-intestinal tract: do they cross the gut-blood barrier?. *Frontiers in Microbiology*.

[61] Zhao L., Luo L., Jia W. (2014). Serum diamine oxidase as a hemorrhagic shock biomarker in a rabbit model. *PLoS One*.

